# Multichannel System Based on a High Sensitivity Superconductive Sensor for Magnetoencephalography

**DOI:** 10.3390/s140712114

**Published:** 2014-07-08

**Authors:** Sara Rombetto, Carmine Granata, Antonio Vettoliere, Maurizio Russo

**Affiliations:** Istituto di Cibernetica “E. Caianiello”, CNR, Pozzuoli, 80078 Naples, Italy; E-Mails: c.granata@cib.na.cnr.it (C.G.); a.vettoliere@cib.na.cnr.it (A.V.); m.russo@cib.na.cnr.it (M.R.)

**Keywords:** superconductivity, SQUIDs, MEG, PACS 74.81.Fa, 85.25.Hv, 07.20.Mc, 85.25.Dq, 87.19.le, 87.85.Ng

## Abstract

We developed a multichannel system based on superconducting quantum interference devices (SQUIDs) for magnetoencephalography measurements. Our system consists of 163 fully-integrated SQUID magnetometers, 154 channels and 9 references, and all of the operations are performed inside a magnetically-shielded room. The system exhibits a magnetic field noise spectral density of approximatively 5 fT/Hz^1/2^. The presented magnetoencephalography is the first system working in a clinical environment in Italy.

## 1. Introduction

Magnetoencephalography (MEG) is a non-invasive, functional imaging technique that measures the magnetic fields generated by the neuronal activity of the brain using ultra high sensitivity magnetic sensors, superconducting quantum inference devices (SQUIDs). Among the available brain imaging methods, MEG uniquely features both a good spatial and an excellent temporal resolution, thus allowing the investigation of key questions in neuroscience and neurophysiology [1–5].

These MEG measurements reflect intracellular electric current flow in the brain and provide direct information about the dynamics of evoked and spontaneous neural activity. MEG measurements are not subject to interferences due to the tissues and fluids lying between the cortex and the scalp [6,7], and magnetic fields are not distorted by the different conduction of the skull, unlike with electroencephalograms (EEGs).

These features make MEG an excellent localization tool for subcortical sources of brain activity and to investigate dynamic neuronal processes, as well as to study cognitive processes, such as language perception, memory encoding and retrieval and higher-level tasks. Moreover, concerning clinical applications [8–10], it has been proven that MEG is a useful diagnostic tool in the identification, prevention and treatment of numerous diseases and illnesses, as it allows the study of brain functions and facilitates the investigation of both spontaneous and evoked activities.

In this paper, we present the MEG system developed by the National Research Council (“Istituto di Cibernetica”) and recently installed in a clinical environment.

## 2. Multichannel System

Multichannel MEG systems take advantage of improvements in SQUID technology, such as reproducibility, compact readout electronics, and coil configuration. With contemporary whole head neuromagnetometers, it is possible to investigate a wide range of phenomena. Meanwhile, artifacts caused by deep brain stimulation can be efficiently suppressed [11]. The MEG system described in this paper is in operation in the Magnetoencephalography Laboratory inside the clinic “Casa di Cura Hermitage s.p.a.” in Naples.

Currently, in Italy, there are only three whole-head MEG systems in operation. One is a commercial system, furnished by Elekta and operating in the Center for Mind/Brain Sciences, a center of the University of Trento [12]. In this system, at each measuring point, there is a pair of planar first-order gradiometers (orthogonal to each other) and an additional magnetometer [13].

The MEG system operating at University of Chieti, inside the Department of Neuroscience and Imaging and in collaboration with the Institute of Advanced Biomedical Technologies [14], is built with magnetometers. A peculiarity of systems built with magnetometers is that they are not only very sensitive to the magnetic field, but also to environmental noise, so that they must operate inside a magnetically-shielded room.

These two MEG systems are mainly used to investigate basic and cognitive neuroscience.

Our MEG system is built with magnetometers, as well, and works inside a magnetically-shielded room. Our system is the only MEG system in Italy currently working in a clinical environment. Moreover, our system presents the possibility to record electrooculogram (EOG) and electrocardiogram (ECG) using additional electrodes. Finally, as our system has been installed in a clinical environment, we have paid special attention to reducing environmental noise.

In the following, we describe in detail our system.

### 2.1. SQUIDs and System Design

SQUIDs are the most sensitive sensors with which to observe changes in magnetic flux. The ultimate sensitivity is reached with low temperature superconductor (low-T*_c_*) SQUIDs working in liquid helium at a temperature of 4.2 K. In these conditions, SQUIDs present an equivalent energy sensitivity that approaches the quantum limit [15]. Due to their unique properties, SQUIDs are widely used in several applications, like biomagnetism, magnetic microscopy [16], non-destructive evaluation [17], geophysics [18], astrophysics [19], quantum information [20] and nanoscience [21–23].

A SQUID sensor is a magnetic flux-voltage converter presenting an extremely low magnetic flux noise. The physical quantities (as, for example, magnetic field, current, voltage, displacements) to be detected are converted in a magnetic flux by using suitable flux transformer circuits [24].

The device consists of a superconducting loop interrupted by two Josephson junctions [24] and uses the properties of flux quantization and the DC Josephson effect to detect very small magnetic fields. To use the SQUID as a magnetometer, it is biased with a current slightly greater than the critical one, so that the SQUID always operates in the so-called “resistive mode”. When these conditions are satisfied, there is a periodic relationship between the voltage across the SQUID and the applied magnetic flux with a period equal to flux quantum Φ_0_ (Φ_0_ = 2.07 × 10^−15^ Wb).

Thus, a SQUID is a flux-to-voltage converter with a non-linear response. If the signals to measure are much smaller than the flux quantum, the SQUID can work in the so-called “small signal mode”. In this configuration, it is flux biased at a voltage-flux characteristic where the responsivity is maximum. Since every continuous signal in a suitable small range around a point can be approximated to a linear signal, the response of the SQUID is proportional to the external magnetic flux. If the signals to detect are greater than Φ_0_, the SQUID response has to be linearized and a flux-locked-loop (FLL) configuration must be used [24]. In this configuration, the output voltage is converted into a current by a resistor and fed back into the SQUID as a flux, via a coil coupled to the sensor, annulling the input magnetic flux. Therefore, the SQUID works as a null detector of magnetic flux, and the voltage across the feedback resistor is proportional to the magnetic flux input. The FLL linearizes the SQUID output, increasing the linear dynamic range. Due to the SQUID's very low output voltage noise, direct voltage readout mode generally leads to a reduction of intrinsic SQUID sensitivity. In order to solve this problem, in recent years, SQUID sensors with a large flux-to-voltage transfer have been proposed to allow a direct-coupled readout scheme without flux modulation [25]. In comparison with the standard electronics, the direct-coupled readout schemes are simpler, more compact, less expensive and very effective.

**Figure 1.**
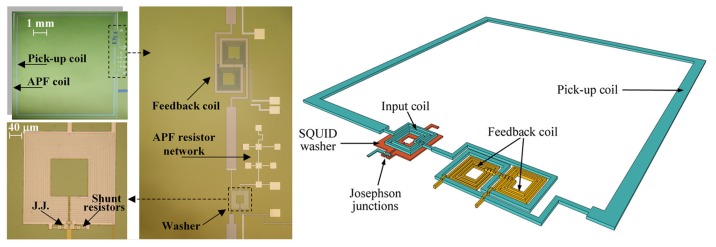
A picture of a superconducting quantum inference device (SQUID) sensor with additional positive feedback and a feedback coil. On the right, a sketch of the SQUID magnetometer showing the main circuit elements is reported.

For the MEG system described here, SQUID sensors have been realized using a standard trilayer technology [26,27] that ensures good performances over time and a good signal-to-noise ratio, even at low frequencies. A detailed fabrication procedure of these SQUID sensors is reported in [28]. Each SQUID magnetometer includes an integrated superconducting flux transformer that works as a magnetic flux pickup to increase the magnetic field sensitivity [29]. The design of the sensor is based on a Ketchen-type magnetometer [30]. The SQUID loop is a square planar washer with an inductance of 260 pH, coupled to a 12-turn thin film input coil with 33 nH inductance connected in series with a square, single turn pickup coil of 64 mm^2^ in area, presenting an inductance of 27 nH. The APF [25] circuit and the feedback coil for FLL configuration [24] are fully integrated on a chip to avoid any additional noise due to an external circuit. In order to obtain a high effective flux-capture area, the mutual inductance between the input coil and the SQUID has been increased by using a large SQUID inductance (L = 250 pH). In such a way, an effective flux capture area of 3 mm^2^, corresponding to a magnetic flux to magnetic field conversion factor of 0.7 nT/Φ_0_, has been obtained [31]. A schematic drawing of the SQUID magnetometer showing the main circuit elements and a picture of the device and its particulars are reported in Figure 1.

**Figure 2.**
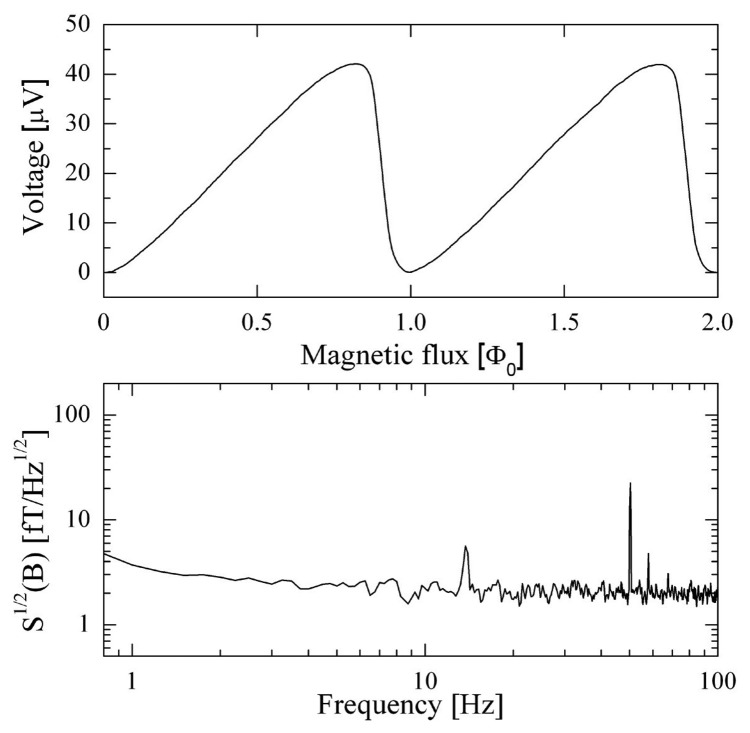
We report here the voltage as a function of the magnetic flux (V-Φ) (**top**) and magnetic field noise spectral density (**bottom**) measured at T = 4.2 K for a SQUID magnetometer. The 1/f corner is less than 3 Hz, which is essentially due to the low frequency noise of the amplifier. The peak at 50 Hz is due to the power line.

For proper operation, SQUIDs have to work at a temperature of 4.2 K, reached by immersing them in liquid helium. In Figure 2, we report an experimental measurement of the voltage as a function of the magnetic flux (V-Φ) for a SQUID magnetometer and its magnetic field noise spectral density, both measured at T = 4.2 K.

The V-Φ has been normalized to Φ_0_, which is equal to 0.7 nT for our SQUID magnetometer. The effect of APF is evident from Figure 2. It renders the V-Φ asymmetric, increasing the voltage responsivity on the stepper side of the characteristic. Thus, for our SQUIDs, it is possible to use simple, direct readout electronics without causing sensor performance degradation.

The magnetic field noise has been measured in FLL by using low noise readout electronics. The noise is essentially independent from the frequency (white noise) for frequencies higher than 10 Hz, and its value is as low as 5 fT/Hz^1/2^. The 1/f corner is less than 3 Hz, which is essentially due to the low frequency noise of the amplifier. The peak at 50 Hz is due to the power line.

**Figure 3.**
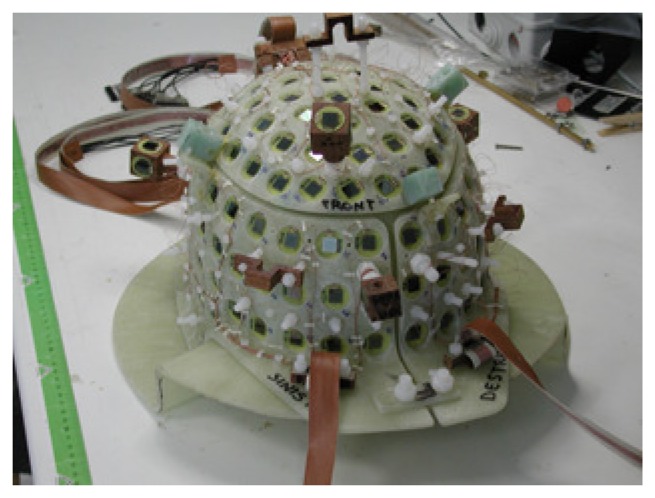
Helmet-shaped array consisting of 163 fully-integrated SQUID magnetometers. The three reference triplets (consisting of 9 SQUIDs) are visible.

The SQUID sensors are arranged over the helmet-shaped array, as reported in Figure 3. This geometry requires a particularly careful wire arrangement and design of the SQUID support to minimize cross-talk [32]. The feedback coil integrated on the SQUID magnetometer chip prevents the cross-talk phenomenon between adjacent channels and allows the integration of a large number of channels [32].

**Figure 4.**
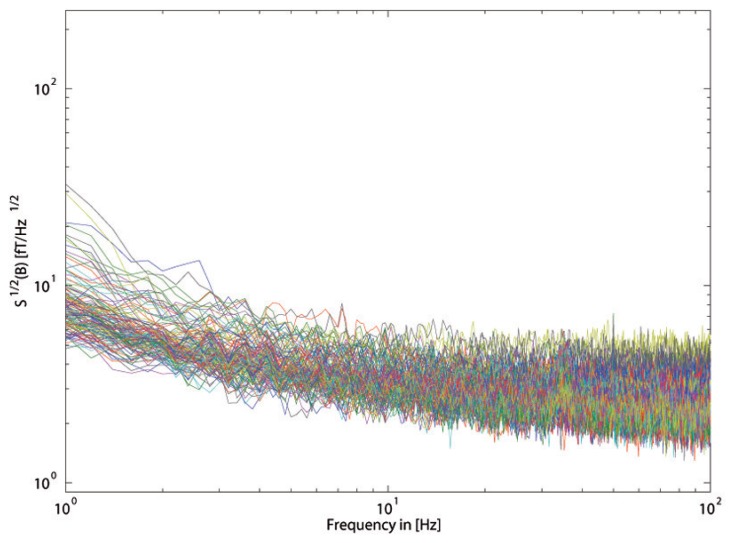
Magnetic field noise spectral density measured at T = 4.2 K for all of the SQUID magnetometers inside the magnetoencephalography (MEG) dewar are reported. For frequencies greater than a few Hz, the noise is essentially independent of the frequency (white noise), and its value is as low as 5 fT/Hz^1/2^.

We have measured the system noise (reported in Figure 4), and it is comparable with the intrinsic noise of a single SQUID sensor.

**Figure 5.**
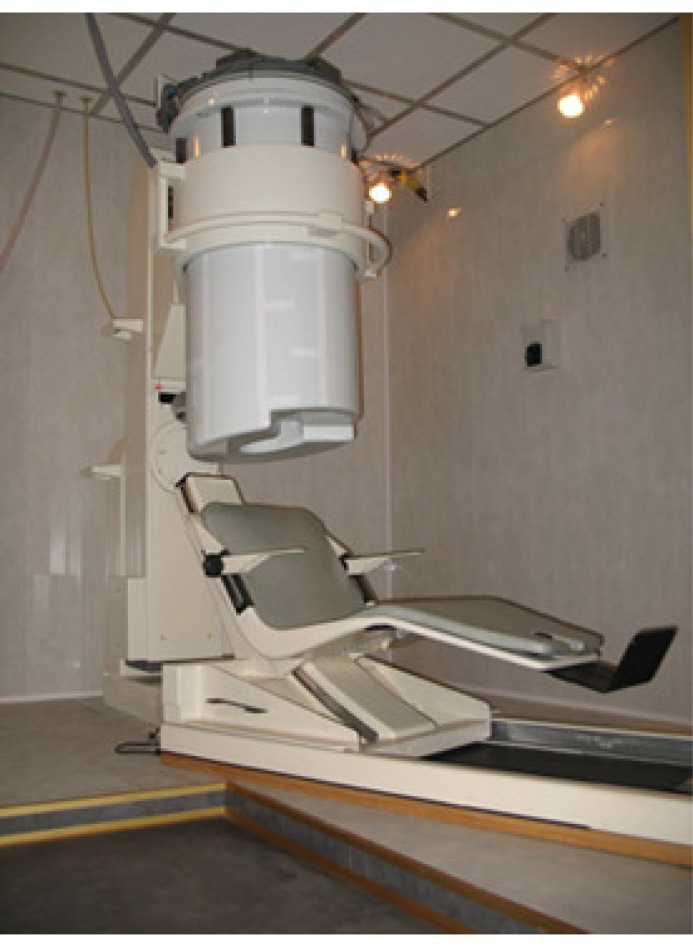
This figure shows the whole MEG system inside the magnetically-shielded room. The dewar is made of fiberglass.

The helmet-shaped array, shown in Figure 3, hosts 154 magnetometer SQUIDs, located so as to ensure that the whole head of the subject is covered and three vector magnetometers (consisting of 9 SQUIDs) located above the helmet and used as references. The MEG system is shown in Figure 5 and consists of 163 fully-integrated SQUID magnetometers.

### 2.2. Technical Equipment

**Figure 6.**
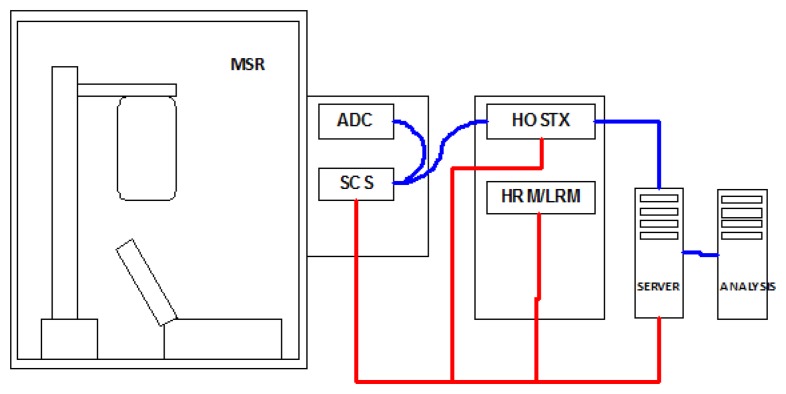
Block diagram of the MEG system. SCS is the sensor control system, PPS is the pre-processing system, HRM and LRM are, respectively, the high resolution monitor and the low resolution monitor.

Continuous data can be acquired simultaneously from the 163 channels in the bandwidth DC-400 Hz. The measured signals are then A/D converted at a sampling frequency of 8.2 kHz and sent to a group of digital signal processors (DSPs). The DSP group is controlled through the console and is used to apply digital filters. Furthermore, it guarantees that all SQUID channels are sampled simultaneously at 1025 Hz with 22-bit ADCs (analog to digital converter). The SQUIDs' parameters can be changed by the operator through the acquisition console. A block diagram of our MEG system is shown in Figure 6. SCS is the sensor controller system, while the PPS is the pre-processing system. HRM and LRM are, respectively, the high resolution monitor and the low resolution monitor. HOSTX indicates computers that contain DSP cards.

The MEG system is also equipped with a 32 integrated non-magnetic EEG-channels cap, with ultra-thin wires, a low profile of sintered Ag/AgCl electrodes, which is optimal for usage inside a MEG helmet [33,34]. This system allows data to be stored digitally and analyzed using both commercial and open-source programs [35]. Scalp EEGs can be inspected visually in real time.

It is also possible to record electrooculograms (EOGs) and electrocardiograms (ECGs) using additional electrodes. Moreover, visual and auditory stimuli and seven external triggers can be used.

### 2.3. Dewar

The dewar enclosing the SQUIDs is an important component and must satisfy strict requirements [3]. In our system, the dewar is made of fiberglass, as this material shows both excellent magnetic properties and optimal thickness. This choice minimizes the distance between the subject's head and SQUIDS, so that the sensors are located only 2 cm above the scalp. Furthermore, to reduce the radiation losses, several layers of mylar have been enclosed inside the inner portion of the dewar.

The dewar has a capacity of 74 L and a helium refill interval of seven days, thanks also to a foam mold that minimizes the heat transfer, as described in Section 2.4.

### 2.4. The System's Thermograms

We have used a thermal imaging camera to detect radiation in the infrared range of the electromagnetic spectrum and to produce thermal images (thermograms) of the MEG system. In fact, as infrared radiation is emitted by all objects above absolute zero, according to the black body radiation law, thermography allows variations in temperature to be identified and measured [36,37]. This analysis demonstrated that the MEG system has excellent thermal resistance. Major variations, within 2 °C, are observed on the joints and in the proximity of the helmet and are due to the reduced thickness of the dewar at those points. When the system is not performing measurements, a foam mold is placed inside the dewar, where usually the head of the subject is placed, to minimize the thermal exchange.

## 3. Magnetically-Shielded Room

### 3.1. Magnetically-Shielded Room: Design

Since the magnetic signals emitted by the human brain are at least eight orders of magnitude smaller than the magnetic disturbances arising from the Earth's magnetic field and urban noise, it is necessary to shield the system from external magnetic signals. The most straightforward noise reduction method is to place the MEG system within a magnetically-shielded room (MSR), which physically reduces environmental noise. MSRs are used all over the world for performing experiments in magnetically quiet environments. Shielded rooms are built by layers of materials providing eddy current shielding (aluminum) and by layers for magnetic shielding (high magnetic permeability materials). The typical shielding factor is 60 dB at 1 Hz and 100 dB at 50 Hz [38]. Their application is compulsory in the field of biomagnetism, where extremely weak magnetic fields originating from the human body are detected.

Our MSR is made of aluminum and *μ*-metal to reduce, respectively, high-frequency and low-frequency noise. Our MSR consists of three nested main layers: a pure aluminum layer (1.5 cm) and two *μ*-metal layers (1.5 mm). Magnetic continuity is maintained by overlay strips. The external dimensions of our MSR are 3.7 × 4.3 × 3.4 m^3^ (l × w × h), and the inner dimensions are 2.9 × 3.7 × 2.9 m^3^. All of the electric connections have been designed to block the introduction of any magnetic noise.

The low-noise dewar and the MSR have allowed us to obtain a noise spectrum of approximately 5 fT/Hz^1/2^, as reported in Figure 4, which is good if compared to the average intrinsic noise of a single SQUID, which is around 2 fT/Hz^1/2^, as shown in Figure 2.

**Figure 7.**
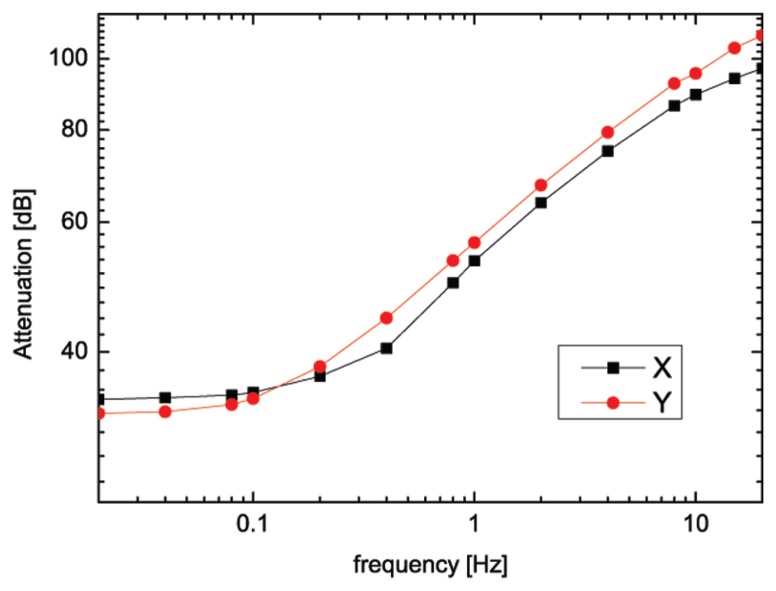
The measured magnetic shielding factor of the magnetically-shielded room to an externally applied field in the frequency range 0.01−20 Hz, as a function of the frequency for the *x* (black line) and *y* (red line) directions. Passive shielding factors of 34.56 dB and 56.29 dB, at 0.1 Hz and 1 Hz, respectively, have been measured.

We also report the response of the MSR to an externally applied field produced by a pair of coils for the *x* (parallel to the length of the MSR) and *y* (parallel to the width of the MSR) component, in the frequency range 0.01−20 Hz (Figure 7). Passive shielding factors, defined as −20Log(B*_int_*/B*_ext_*), have been measured. For our MSR, shielding factors are 34.56 dB at 0.1 Hz and 56.29 dB at 1 Hz, where *B_int_* is the magnetic field measured inside the MSR and B*_ext_* is the magnetic field measured outside the MSR. It has been observed also that the attenuation in the *y* direction is slightly lower than in the *x* direction, probably due to the aluminum structure that sustains the door of the MSR.

### 3.2. Anti-Vibration Pads

Small movements or vibrations of the dewar that contain the SQUIDS could introduce a large time-variance in the recorded MEG signal. For this reason, we placed Dynemech DNM Foundation Isolation Plates [39] below the MSR. Filler foam has been placed in the gaps between the insulation plates. These plates are specifically designed to provide low frequency vibration isolation and can bear a load in the range of 1–4 Kg/cm^2^.

## 4. Noise Reduction Algorithm and Preliminary Measurements

Despite the MSR and despite the use of anti-vibration plates, it was possible to observe the effects of some residual environmental noise, especially at low frequencies. Thus, a noise reduction algorithm (NRA) was implemented to improve the signal-to-noise ratio.

MEG signals and reference signals can be described as:

(1) $$\begin{array}{cc} s_{i}(t) & \text{MEG channels (}i=1,\ldots \;154\text{)} \end{array}$$

(2) $$\begin{array}{cc} \rho_{j}(t) & \text{reference channels (}j=1,\ldots 9\text{)} \end{array}$$

As is well known, an unprocessed signal is assumed to be a superposition of signal and undesired noise. Here, we assume that reference channels measure “only” the background field. For each sensor, the corrected signal, *c_i_*, can be expressed in terms of raw sensor output, *s_i_*, and a linear superposition of reference outputs, *ρ_j_*, as:

$$c_{i}=s_{i}-\sum_{j=1}^{9}\tau_{j}\rho_{j}$$

where *τ_j_* are the weighting coefficients [40] calculated using single value decomposition (SVD) [41] on reference data. This is a robust method for decomposing the data matrix into signal and noise subspaces and has been shown to be successful in removing a variety of noise and artifacts from MEG data [42].

**Figure 8.**
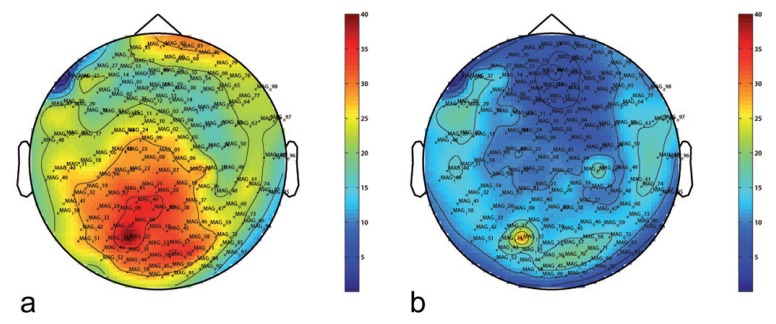
Topographic distribution over the head of the power-spectrum in the range 10–12 Hz before (**a**) and after (**b**) the application of the noise-reduction algorithm (for a subject recorded in resting state with open eyes).

After NRA application, the residual noise is very close to the intrinsic noise for SQUIDs. NRA has been successful also in removing the 50 Hz noise due to the power line (Figure 8).

In order to verify the operativeness of the MEG system, we report some preliminary measurements performed on some healthy voluntary subjects. We recorded and analyzed MEG data in two conditions: open eyes and closed eyes.

Here, we report recorded signals after the preprocessing routine, which is necessary to reduce environmental interference and biological noise, and the NRA application. Identification of artifact-contaminated epochs has been carried out within the Fieldtrip toolbox [35]. We bandpass-filtered recorded data between 2 and 100 Hz and applied a notch filter at 50 Hz.

**Figure 9.**
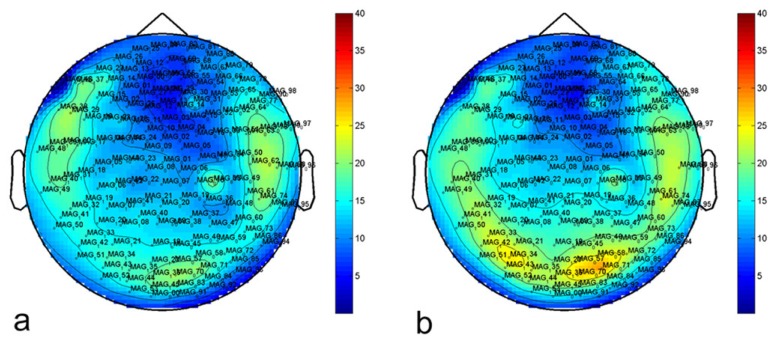
Topographic distribution over the head of the power-spectrum in the range 10–12 Hz after the application of the noise-reduction algorithm for a subject recorded in resting state with open eyes (**a**) and closed eyes (**b**) (alpha rhythm).

In Figure 9, we report the topographic distribution over the head of the power-spectrum in the range of 10–12 Hz. In Figure 9a, measurements performed on a subject with open eyes are reported. In Figure 9b, measurements on the same subject with closed eyes are reported. As expected, we recorded major activation in the occipital region when the subject was recorded in resting state with closed eyes (alpha rhythm).

During last 40 years, MEG has been acknowledged as a very important tool for basic and clinical neuroscience. We plan to perform different kind of experiments, in the framework of several collaborations. As this system is placed inside a clinic, we plan to perform also experiments on clinical patients, with special care for neurodegenerative disorders (like, for example, Alzheimer, Parkinson, *etc.*) and autism in children. We plan also to integrate our results with fMRI results.

## 5. Conclusions

We have developed an ultra-low noise biomagnetic multi-channel system, which operates reliably in a clinical environment. We have demonstrated the main characteristics, technical equipment and performance of our MEG system, which consists of 163 full- integrated SQUIDS, developed at Istituto di Cibernetica of the National Research Council and located in a clinical environment.

In fact, this MEG system presents good characteristics for clinical and cognitive research. The noise floor is about 5 fT/Hz^1/2^, and sensor performances are stable during operation, so that high-quality MEG recordings are possible with this system. We would like to stress that there are only three other MEG systems currently operating in Italy and that the presented MEG is the first system to be in operation in a clinical environment in Italy.
